# Zebras Também Existem!

**DOI:** 10.36660/abc.20230834

**Published:** 2024-03-04

**Authors:** Murillo de Oliveira Antunes

**Affiliations:** 1 Universidade São Francisco Bragança Paulista SP Brasil Universidade São Francisco (USF), Bragança Paulista, SP - Brasil; 2 Hospital Universitário São Franscisco de Assis na Providência de Deus Bragança Paulista SP Brasil Hospital Universitário São Franscisco de Assis na Providência de Deus, Bragança Paulista, SP - Brasil

**Keywords:** Doença de Fabry/complicações, Doenças do Recém-Nascido, Hipertrofia Ventricular Esquerda, Cardiomiopatia Hipertrófica/diagnóstico por imagem, Terapia de Reposição Enzimática, Doenças Raras

A doença de Fabry (DF) é uma doença rara de armazenamento lisossômico hereditário ligado ao cromossomo X, causado pela atividade deficiente da enzima alfa-galactosidase A que leva ao acúmulo de globotriasilceramida (Gb3) nos tecidos afetados, incluindo o coração, cérebro e rins.^1^ Na DF a lesão cardíaca inicia-se cedo progredindo de forma subclínica antes do aparecimento de sintomas e geralmente se manifesta como hipertrofia ventricular esquerda simulando cardiomiopatia hipertrofia (CMH).^2^ Estudos recente demonstraram uma prevalência de DF até 5% em pacientes que tinham como diagnóstico inicial CMH.^3^ Após a introdução da terapia de reposição enzimática, a DF merece atenção especial no diagnóstico diferencial nos pacientes com hipertrofia ventricular inexplicada e CMH, pois é uma cardiopatia que apresenta tratamento específico, porém o diagnóstico e tratamento precoce são fundamentais para prevenir a progressão da doença reduzindo as taxas de eventos cardiovasculares.^4-6^

Com o objetivo de investigar as diferenças clínicas e de exames entre CMH e DF, o estudo de Akhan et al.,^7^ avaliou de forma retrospectiva 60 pacientes com CMH e 40 com DF. Nessa população, com idade média semelhante, os autores evidenciaram, de maneira geral, que as características das duas condições cardíacas compartilham mais semelhanças que diferenças, tornando o diagnóstico um desafio na prática clínica.

Os autores indicaram que o grupo com CMH apresenta alterações cardíacas estrutural e funcionais mais significativas, resultando em sintomatologia mais pronunciada e na necessidade de intervenções medicamentosa.^7^ Estes achados corroboram o mecanismo fisiopatológico implicado na origem das duas doenças, enquanto na CMH as mutações nos genes do sarcômero cardíaco desencadeiam hipertrofia, desarranjo e fibrose, na DF o depósito de Gb3 no cardiomiócitos ativam mecanismos inflamatórios e neuro-hormonais envolvidos na formação hipertrofia e fibrose tecidual, embora de forma indireta e em menor magnitude.^8^ A diminuição da taxa de filtração glomerular foi mais significativa na DF, visto que o comprometimento renal ocorre de maneira progressiva e precoce, no entanto é importante destacar, que nesse contexto do diagnóstico diferencial, é um achado que deve ser cuidadosamente considerado.^9^ Os achados do ECG indicaram ter pouco valor para a diferenciação entre as duas cardiopatias. Algo relevante a ser considerado foi o intervalo QT, menor no grupo de DF, sugerindo que esta medida pode ser mais relevante que a redução do intervalo PR (PQ) para diagnóstico de DF, como também demonstrado por Namdar et al.,^10^

*"Quando escutar batidas de casco, pense em cavalos, não em zebras"* (Dr. Theodore Woodward 1940), mas lembrem-se: zebras também existem! No desafiador cenário das doenças raras, como DF, negligenciar a possibilidade desse diagnóstico pode resultar em atrasos e erros diagnósticos reduzindo os benefícios do tratamento específico. Portanto uma história boa detalhada, especialmente com realização do heredograma familiar, juntamente com exame físico minuciosos e reconhecimento das diferenças (muitas vezes sutis) nos exames complementares são únicas pegadas que nos levam ao encontro das zebras.

## References

[1] Pieroni M, Moon JC, Arbustini E, Barriales-Villa R, Camporeale A, Vujkovac AC (2021). Cardiac Involvement in Fabry Disease: JACC Review Topic of the Week. J Am Coll Cardiol.

[2] Ommen SR, Nishimura RA, Edwards WD (2003). Fabry disease: a mimic for obstructive hypertrophic cardiomyopathy?. Heart.

[3] Fernandes F, Antunes MO, Hotta VT, Rochitte CE, Mady C (2019). Doenças de Depósito como Diagnóstico Diferencial de Hipertrofia Ventricular Esquerda em Pacientes com Insuficiência Cardíaca e Função Sistólica Preservada. Arq Bras Cardiol.

[4] Ortiz A, Germain DP, Desnick RJ, Politei J, Mauer M, Burlina A (2018). Fabry disease revisited: management and Treatment recommendations for adult patients. Mol Genet Metab.

[5] Linhart A, Germain DP, Olivotto I, Akhtar MM, Anastasakis AM, Hughes D (2020). An expert consensus document on the management of cardiovascular manifestations of Fabry disease. Eur J Heart Fail.

[6] Germain DP, Elliott PM, Falissard B, Fomin VV, Hilz MJ, Jovanovic A (2019). The effect of enzyme replacement therapy on clinical outcomes in male patients with Fabry disease: a systematic literature review by a European panel of experts. Mol Genet Metab Rep.

[7] Akhan O, Kis M, Güzel T, Zoghi M (2024). Differences Between Two Distinct Hypertrophic Cardiac Conditions: Fabry Disease versus Hypertrophic Cardiomyopathy. Arq Bras Cardiol.

[8] Yousef Z, Elliott PM, Cecchi F, Escoubet B, Linhart A, Monserrat L (2013). Left ventricular hypertrophy in Fabry disease: a practical approach to diagnosis. Eur Heart J.

[9] Del Pino M, Andrés A, Bernabéu AÁ, de Juan-Rivera J, Fernández E, de Dios García Díaz J (2018). Fabry Nephropathy: An Evidence-Based Narrative Review. Kidney Blood Press Res.

[10] Namdar M, Steffel J, Jetzer S, Schmied C, Hürlimann D, Camici GG (2012). Value of electrocardiogram in the differentiation of hypertensive heart disease, hypertrophic cardiomyopathy, aortic stenosis, amyloidosis, and Fabry disease. Am J Cardiol.

