# Supplementary material for: Zebras Também Existem!
Source: Arq Bras Cardiol. 2024 Mar 4;121(1):e20230834. [Article in Portuguese] doi: 10.36660/abc.20230834 (PMC11081203; doi:10.36660/abc.20230834)
Supplement: Translation

# Translation: Zebras Exist, Too!

**Authors:** Murillo de Oliveira Antunes

**Affiliations:** 1 Bragança Paulista SP Brazil Universidade São Francisco (USF), Bragança Paulista, SP - Brazil; 3 Bragança Paulista SP Brazil Hospital Universitário São Franscisco de Assis na Providência de Deus, Bragança Paulista, SP - Brazil

**Keywords:** Fabry Disease/complications, Infant, Newborn, Diseases, Hypertrophy, Left Ventricular, Cardiomyopathy, Hypertrophic/diagnostic, imaging, Enzyme Replacement Therapy, Rare Disease

Fabry disease (FD) is a rare X-linked hereditary lysosomal storage disease caused by deficient activity of the enzyme alpha-galactosidase A that leads to the accumulation of globotriasylceramide (Gb3) in affected tissues, including the heart, brain, and kidneys.^1^ In FD, cardiac damage begins early and progresses subclinically before the appearance of symptoms and generally manifests as left ventricular hypertrophy simulating hypertrophic cardiomyopathy (HCM).^2^ Recent studies have demonstrated a prevalence of FD of up to 5% in patients whose initial diagnosis was HCM.^3^ After the introduction of enzyme replacement therapy, FD deserves special attention in the differential diagnosis in patients with unexplained ventricular hypertrophy and HCM, as it is a heart disease that requires specific treatment. However, early diagnosis and treatment are essential to prevent the progression of the disease by reducing cardiovascular event rates.^4-6^

With the aim of investigating the clinical and exam differences between HCM and DF, the study of Akhan et al.^7^ retrospectively evaluated 60 patients with HCM and 40 with FD. In this population, with a similar average age, the authors showed, in general, that the characteristics of the two cardiac conditions share more similarities than differences, making diagnosis a challenge in clinical practice.

The authors indicated that the HCM group presents more significant structural and functional cardiac changes, resulting in more pronounced symptoms and the need for drug interventions.^7^ These findings corroborate the pathophysiological mechanism implicated in the origin of both diseases, while in HCM mutations in cardiac sarcomere genes trigger hypertrophy, disarray, and fibrosis, in FD, the deposition of Gb3 in cardiomyocytes activate inflammatory and neurohormonal mechanisms involved in the formation of hypertrophy and tissue fibrosis, although indirectly and to a lesser extent.^8^ The decrease in the glomerular filtration rate was more significant in FD since renal impairment occurs progressively and early; however, it is important to highlight that in this context of diagnosis differential, it is a finding that must be carefully considered.^9^ The ECG findings indicated that they have little value for differentiating between the two heart diseases. Something relevant to be considered was the QT interval, which was shorter in the FD group, suggesting that this measure may be more relevant than reducing the PR interval (PQ) for diagnosing FD, as also demonstrated by Namdar et al.^10^

*"When you hear hoofbeats, think of horses, not zebras."* (Dr. Theodore Woodward 1940), but remember: zebras exist too! In the challenging scenario of rare diseases such as FD, neglecting the possibility of this diagnosis can result in delays and diagnostic errors, reducing the benefits of specific treatment. Therefore, a good detailed history, especially with the family pedigree, together with a thorough physical examination and recognition of differences (often subtle) in complementary exams, are the only steps that lead us to meet the zebras.
